# Myalgic Encephalomyelitis/Chronic Fatigue Syndrome: When Suffering Is Multiplied

**DOI:** 10.3390/healthcare9070919

**Published:** 2021-07-20

**Authors:** Anthony L. Komaroff

**Affiliations:** Division of General Medicine, Department of Medicine, Brigham & Women’s Hospital, Harvard Medical School, 1620 Tremont, Boston, MA 02120, USA; komaroff@hms.harvard.edu

**Keywords:** myalgic encephalomyelitis/chronic fatigue syndrome, etiology, diagnostic testing

## Abstract

Myalgic encephalomyelitis/chronic fatigue syndrome (ME/CFS) is an illness defined predominantly by symptoms. Routine laboratory test results often are normal, raising the question of whether there are any underlying objective abnormalities. In the past 20 years, however, new research technologies have uncovered a series of biological abnormalities in people with ME/CFS. Unfortunately, many physicians remain unaware of this, and some tell patients that “there is nothing wrong” with them. This skepticism delegitimizes, and thereby multiplies, the patients’ suffering.

The symptoms caused by any illness should be suffering enough. Yet, with some illnesses, the suffering often is multiplied by skepticism about the illness. That is the case with myalgic encephalomyelitis/chronic fatigue syndrome (ME/CFS).

In an article in *Healthcare,* Whitney Dafoe—who has been diagnosed with ME/CFS—describes his experience with an extremely severe form of the illness [1]. He describes the physical and mental crashes and the extreme sensitivity to any kind of sensory input. He also describes the isolation, the loss, the complete and sudden disruption in the life of a young adult, a life that was on the runway and cleared for takeoff.

Why have some physicians and biomedical scientists been skeptical about the “legitimacy” of ME/CFS? Primarily, it is because the illness has been defined largely by symptoms. Since it is difficult for symptoms to be confirmed objectively, physicians have sought objective laboratory evidence of underlying biological abnormalities—abnormalities that an individual cannot simply imagine, abnormalities that could explain the symptoms. Initially, that proved hard.

When interest in this condition was renewed in the mid-1980s, there was little such evidence: the “standard” laboratory tests ordered by physicians—typically, tests of red and white blood cells, a battery of about 20 chemistry tests, and a urinalysis—produced normal results. That posed a problem for the physicians. Their patients were suffering, and it was their job to make a diagnosis and prescribe a treatment, but the standard test results were normal: the physicians did not have a diagnosis.

At this point, the physicians had several options. First, they could have entertained some new hypotheses about what was causing the symptoms, and ordered new types of tests. Second, they could have said: “I just can’t figure out what’s making you sick, and don’t know how to help you.” Third, although they could not determine the diagnosis, they could have prescribed a treatment that might improve the symptoms even if they were not really sure what had caused the symptoms. That happens every day in the practice of medicine. For example, there is no diagnostic test for migraine headaches, yet doctors make that diagnosis every day based just on a combination of symptoms, and do not dispute the validity of the illness because there is no diagnostic test.

Unfortunately, the normal results of “standard” laboratory tests led some physicians to choose a fourth option: to conclude that there were no underlying biological abnormalities causing the symptoms. Even though the physicians knew that the “standard” tests they had ordered represented only a tiny fraction of all of the tests available to them, the normal results of that tiny fraction were enough for them to render a judgment. It was a harsh judgment: “There is nothing wrong with you.”

For these physicians, it was an efficient solution: it transformed what had been their problem—the lack of a diagnosis they were expected to make—into their patient’s problem. When the patients were told, implicitly or explicitly, that their symptoms were imaginary, it multiplied the suffering.

And then these skeptical physicians also conveyed their judgments, implicitly or explicitly, to the patients’ families, friends and employers. The doctors’ judgment led these people—the people who were most important in the patients’ lives—to wonder whether the patients’ suffering was legitimate. That further multiplied the suffering.

There was always an obvious alternative conclusion to the judgment that “there is nothing wrong with you”: the standard laboratory tests might simply have been measuring the wrong things. Yet that alternative conclusion was ignored.

Since the resurgence of interest in ME/CFS 35 years ago, whole new technologies have become available that allow physicians and biomedical scientists to study human biology in ways that previously were not possible, e.g., noninvasive techniques for imaging the anatomy and physiology of the brain; polymerase chain reaction diagnostics; rapid nucleic acid sequencing; techniques for measuring gene expression; the ability to measure simultaneously thousands of molecules in a single sample (the “omics” revolution); metagenomic studies of the microbiome, and recognition of the impact of the microbiome on human health. In fact, these and other technologies have revealed things that the standard laboratory tests cannot—abnormalities that previously were invisible to doctors.

In 2015 the U.S. National Academy of Medicine (NAM) reviewed a literature of over 9000 publications on ME/CFS, and concluded that it was a “serious, chronic, complex systemic disease” [2]. The NAM estimated that in the U.S., alone, 836,000 to 2.5 million people suffer from ME/CFS [2], making it somewhat more common than multiple sclerosis [3].

A large literature now describes multiple underlying biological abnormalities in people with ME/CFS. Some of the evidence comes from tests that have been available for decades but are not part of the “standard” laboratory test battery [4], and some evidence comes from the new technologies mentioned above. Unfortunately, many physicians are unaware of the new discoveries about ME/CFS.

The abnormalities all converge on and can affect the brain, and fall into five categories. First, there are anatomic, physiologic and electrical abnormalities in the brain [5]. Second, various elements of the immune system are chronically activated and in some people those elements are exhausted—perhaps secondary to years of chronic activation [5]. This includes chronic activation of the brain’s innate immune system—neuroinflammation [6]. It also includes evidence of autoimmunity, including autoantibodies directed at targets in the central and autonomic nervous system [7]. Third, there also is evidence of impaired energy metabolism: the person with ME/CFS feels he or she lacks “energy” because his or her cells have a reduced ability to generate energy molecules (adenosine triphosphate, or ATP) [8]. Along with the abnormalities in energy metabolism, there is associated oxidative stress, or redox imbalance [8]. Fourth, the autonomic nervous system is dysregulated, one consequence of which appears to be impaired blood flow to the brain [9]. Fifth, there are characteristic abnormalities of the gut microbiome [10], with increased numbers of pro-inflammatory bacterial species and decreased numbers of butyrate-producing anti-inflammatory species.

What remains unclear are the mechanistic details as to how the abnormalities in each of these five categories affect each other, and whether one of them is the initial and primary abnormality [5,8]. In this next decade, the growing community of global investigators who are studying ME/CFS should place a high priority on refining our understanding of each of these categories of abnormality, and an even higher priority on understanding how they are connected. This is essential for developing good diagnostic tests, and effective treatments.

Whitney Dafoe ends the description of his suffering by emphasizing the silver lining around the cloud that he has lived with for nearly 20 years. He says he has learned a great deal about what is important in life, and that “ME/CFS is the greatest teacher I’ve ever had.”

I would like to think that ME/CFS will also prove to be a great teacher to the growing community of physicians and biomedical investigators involved in caring for and studying the illness. In particular, I speculate that the connections between the various abnormalities involving the central and autonomic nervous system, immune system, energy metabolism, redox imbalance, and the human microbiome that have been noted in ME/CFS will prove to be central also to the pathophysiology of many other diseases.

In particular, the COVID-19 pandemic appears to be producing millions of new cases of an ME/CFS-like condition [11], and NIH has allocated more than $1 billion to study this and other post-COVID chronic illnesses. Hopefully, this investment will produce more answers.

Of the personal lessons that I, as a physician, have learned from ME/CFS, perhaps the most important is that, if patients tell you they are suffering, your default assumption should be to believe them—even if you cannot find an answer with the diagnostic technology you first deploy. Above all, never succumb to the temptation to dismiss the patient’s symptoms because you cannot explain them. That may ease your anxiety, but it only multiplies the patient’s suffering.

## Data Availability

Not applicable.

## References

[1] Dafoe W. (2021). Extremely severe ME/CFS-A personal account. Healthcare.

[2] Institute of Medicine (2015). Beyond Myalgic Encephalomyelitis/Chronic Fatigue Syndrome: Redefining an Illness.

[3] Wallin M.T., Culpepper W.J., Campbell J.D., Nelson L.M., Langer-Gould A., Marrie R.A., Cutter G.R., Kaye W.E., Wagner L., Tremlett H. (2019). The prevalence of MS in the United States. Neurology.

[4] Bates D.W., Buchwald D., Lee J., Kith P., Doolittle T., Rutherford C., Churchill W.H., Schur P., Wener M., Wybenga D. (1995). Clinical laboratory test findings in patients with chronic fatigue syndrome. Arch. Intern. Med..

[5] Komaroff A.L., Lipkin W.I. (2021). Insights from myalgic encephalomyelitis/chronic fatigue syndrome may help unravel the pathogenesis of post-acute COVID-19 syndrome. Trends Mol. Med..

[6] Nakatomi Y., Mizuno K., Ishii A., Wada Y., Tanaka M., Tazawa S., Onoe K., Fukuda S., Kawabe J., Takahashi K. (2014). Neuroinflammation in patients with chronic fatigue syndrome/myalgic encephalomyelitis: An ^11^C-(R)-PK11195 PET study. J. Nucl. Med..

[7] Sotzny F., Blanco J., Capelli E., Castro-Marrero J., Steiner S., Murovska M., Scheibenbogen C. (2018). Myalgic encephalomyelitis/chronic fatigue syndrome—Evidence for an autoimmune disease. Autoimmun. Rev..

[8] Paul B.D., Lemle M.D., Komaroff A.L., Snyder S.H. (2021). Redox imbalance links COVID-19 and myalgic encephalomyelitis/chronic fatigue syndrome. Proc. Natl. Acad. Sci. USA.

[9] Van Campen C.L.M.C., Rowe P.C., Visser F.C. (2020). Cerebral blood flow is reduced in severe myalgic encephalomyelitis/chronic fatigue syndrome patients during mild orthostatic stress testing: An exploratory study at 20 degrees of head-up tilt testing. Healthcare.

[10] Giloteaux L., Goodrich J.K., Walters W.A., Levine S.M., Ley R.E., Hanson M.R. (2016). Reduced diversity and altered composition of the gut microbiome in individuals with myalgic encephalomyelitis/chronic fatigue syndrome. Microbiome.

[11] Komaroff A.L., Bateman L. (2021). Will COVID-19 lead to myalgic encephalomyelitis/chronic fatigue syndrome?. Front. Med..

